# Heterogeneity of neutrophils and inflammatory responses in patients with COVID-19 and healthy controls

**DOI:** 10.3389/fimmu.2022.970287

**Published:** 2022-11-16

**Authors:** Jintao Xu, Bing He, Kyle Carver, Debora Vanheyningen, Brian Parkin, Lana X. Garmire, Michal A. Olszewski, Jane C. Deng

**Affiliations:** ^1^ Research Service, LTC Charles S. Kettles Veterans Affairs Medical Center, Department of Veterans Affairs Health System, Ann Arbor, MI, United States; ^2^ Division of Pulmonary and Critical Care Medicine, Department of Internal Medicine, University of Michigan Medical School, Ann Arbor, MI, United States; ^3^ Department of Computational Medicine and Bioinformatics, University of Michigan, Ann Arbor, MI, United States; ^4^ Division of Hematology and Oncology, Department of Internal Medicine, University of Michigan Health System, Ann Arbor, MI, United States

**Keywords:** COVID-19, immune response, single-cell sequencing, neutrophil heterogeneity, SARS – CoV – 2

## Abstract

Severe respiratory viral infections, including SARS-CoV-2, have resulted in high mortality rates despite corticosteroids and other immunomodulatory therapies. Despite recognition of the pathogenic role of neutrophils, in-depth analyses of this cell population have been limited, due to technical challenges of working with neutrophils. We undertook an unbiased, detailed analysis of neutrophil responses in adult patients with COVID-19 and healthy controls, to determine whether distinct neutrophil phenotypes could be identified during infections compared to the healthy state. Single-cell RNA sequencing analysis of peripheral blood neutrophils from hospitalized patients with mild or severe COVID-19 disease and healthy controls revealed distinct mature neutrophil subpopulations, with relative proportions linked to disease severity. Disruption of predicted cell-cell interactions, activated oxidative phosphorylation genes, and downregulated antiviral and host defense pathway genes were observed in neutrophils obtained during severe compared to mild infections. Our findings suggest that during severe infections, there is a loss of normal regulatory neutrophil phenotypes seen in healthy subjects, coupled with the dropout of appropriate cellular interactions. Given that neutrophils are the most abundant circulating leukocytes with highly pathogenic potential, current immunotherapies for severe infections may be optimized by determining whether they aid in restoring an appropriate balance of neutrophil subpopulations.

## Introduction

Severe lung injury and systemic inflammation are the main hallmarks of severe respiratory viral infections, including SARS-CoV-2 (1). Neutrophils, or polymorphonuclear leukocytes, are the most abundant leukocyte population in the blood and found in high numbers in the lung during severe respiratory viral infections (2). During viral infections, neutrophils can contribute to viral clearance through mechanisms such as phagocytosis, and release of neutrophil extracellular traps, secretion of cytokines and activation of the adaptive immune response (2–4). However, the overactivation of neutrophils may cause bystander damage to host tissues and lead to poor outcomes (2–4). Although neutrophils are considered as a primary cellular driver of the pathogenesis of acute respiratory distress syndrome (ARDS) and have been implicated in the pathophysiology of severe COVID-19 (5–9), the challenges of isolating neutrophils for analysis and their inability to survive cryopreservation have resulted in poor understanding of their function in disease progression (10). Additionally, neutrophils are still largely considered to be a homogenous effector cell population with clearly established roles in combatting bacterial and fungal infections, but their mechanistic contributions to the immunopathogenesis or clearance of viral infections remain unclear. While recent studies have started to recognize the heterogeneity of neutrophils during cancer and other chronic diseases (11, 12), whether different neutrophil subtypes exist beyond “immature,” “mature,” and “senescent” is uncertain during an acute infection. The goal of our study, therefore, was to test the hypothesis that different phenotypes of mature neutrophils exist under basal healthy conditions, with subsequent changes in relative abundance during acute respiratory viral infections depending on the severity of immunopathology of COVID-19 disease.

To investigate this, we employed single-cell RNA sequencing (scRNA-seq) analysis of the peripheral immune response to SARS-CoV-2, a technique that has provided novel insights into immune cell heterogeneity and dysregulation during COVID-19 (7, 10, 13–18). Most of the studies to date have utilized preserved peripheral blood mononuclear cells (PBMC), which are mainly comprised of monocyte and lymphocyte populations. As a result, the literature largely reflects an incomplete and possibly biased picture of increased immature and dysfunctional neutrophils – largely reflecting low-density neutrophils that are captured in the PBMC fraction (15, 19, 20). Given that neutrophils are the most abundant leukocyte in peripheral blood during both health and infection, a comprehensive analysis of unpreserved, fresh neutrophils from human subjects is needed in order to determine whether mature neutrophils have the ability to adopt distinct phenotypes, and how these phenotypes change during clinically significant respiratory infections such as SARS-CoV-2.

In this study, we recruited adult subjects hospitalized with mild or severe SARS-CoV-2 infections, in order to examine how neutrophil phenotypes changed based upon the severity of infection over time, compared to healthy adults. We took care only to include adult patients who were without significant co-morbidities or underlying immunosuppressive conditions that might confound neutrophil phenotypes. Our analysis resulted in the identification of 7 mature and 2 immature neutrophil clusters, which had differential pathway activation patterns. Our results also demonstrated that quantitatively and qualitatively, neutrophils are a potentially more robust cellular regulator of inflammatory responses than monocytes, further underscoring the importance of investigating the considerable heterogeneity of responses in the neutrophil population.

## Methods

### Patient cohort, biological samples, and preparation of single-cell suspensions

A total of 11 patients admitted with SARS-CoV-2 infections and 5 healthy control subjects (all outpatients) were enrolled from the Veterans Affairs Ann Arbor Healthcare System and Michigan Medicine University Hospital. Between June, 2020 and June, 2021, all adult patients admitted with COVID-19 disease to these two facilities were screened and recruited if they had no evidence of baseline immunosuppression (e.g., chronic prednisone use of 15 mg or more daily, other immunosuppressive medications, HIV infection, neutropenia), active or recent malignancy within past 5 years, immunomodulatory therapies (e.g., biologics), chronic infection (e.g., hepatitis viral infection), significant systemic autoimmune disease, chronic malnutrition or debility, significant chronic organ dysfunction (e.g., chronic liver disease, poorly controlled diabetes, severe COPD or lung disease, heart failure with EF<40%), chronic alcohol consumption of >5 drinks a day, or active co-infection other than SARS-CoV-2. 11 patients with COVID-19 were classified into two groups based upon severity – “mild” (n = 4, hospitalized but needing <50% O2), or “severe” (n = 7, hospitalized but needing > 50% O2 or in Intensive Care Unit). Out of seven patients with severe symptoms, 4 patients deteriorated clinically and passed away. The demographic and disease characteristics of the prospectively recruited patients studied by scRNA-seq are listed in **Supplementary Figure 1**. All participants had to be capable of providing written informed consent for sample collection and subsequent analyses.

Within 72 hours of hospital admission (“T1”) and 5-7 days later (“T2), blood was collected into lavender top tubes (EDTA) and processed immediately after collection except that one patient was sampled day 15 days later. Data from this patient passed our strict quality control and clustered with the other patients within the same group. Healthy control subjects and 2 Veterans who were discharged did not get a second sample. For PMBC and granulocyte isolation from patient blood, 5ml of blood was carefully layered on 5ml of Lymphocyte-poly isolation media (NC9950836, Fisher) in a 15ml conical tube in a biosafety cabinet. The sample was spun in a sealed bucket at room temperature, 500g, 35 minutes. After centrifuge, leukocyte bands containing mononuclear cells and granulocytes were transferred to another 50 ml conical tube. Cells were diluted with an equal volume of HBSS without Calcium and Magnesium and spun at room temperature, 350g for 10min. The supernatant was removed from each pellet and suspended in 5ml 1x ACK lysis buffer for 2 minutes. Pellets were suspended with PBS containing 0.04% BSA and then counted. An aliquot of mononuclear and granulocyte cells was processed in a cytospin and stained using Diff Quick (26096-25, Electron Microscopy Sciences). Cell differentials were counted, and the mononuclear and granulocyte populations were combined to achieve a 1:1 ratio. Cells were immediately processed for the single-cell RNA library. For all samples, cell viability exceeded 90%.

### Single-cell RNA library preparation and sequencing

The scRNA-seq libraries were constructed using a Chromium Next GEM Single Cell 3ʹ Reagent Kit v3.1 (10X Genomics) according to the manufacturer’s introduction. In brief, the cell suspension (700-1,200 cells per ul) was loaded onto a Chromium single cell controller to generate single-cell gel beads in the emulsion (GEMs). Following this, scRNA-seq libraries were constructed according to the manufacturer’s instructions. The libraries were sequenced using an Illumina Novaseq sequencer at Advanced Genomics Core of the University of Michigan using the suggested cycling from 10X Genomics.

### Single-cell RNA-seq data processing

We aligned single-cell RNA sequencing data against the GRCh38 human reference genome and preprocessed using cellranger pipeline (version 6.0.0). A preliminary single-cell gene expression matrix was then exported from cellranger for further analysis. Quality control was applied to cells based on three metrics - the total UMI counts, number of detected genes, and proportion of mitochondrial gene counts per cell. Specifically, cells with less than 500 UMI counts and 200 detected genes were filtered out, as well as cells with more than 20% mitochondrial gene counts. Thereafter, we applied DoubletFinder, which identifies doublets formed from transcriptionally distinct cells (21), to remove potential doublets. The expected doublet rate was set to be 0.075, and cells predicted to be doublets were filtered out. After quality control, a total of 108,597 cells were collected for further analysis.

### Clustering and cell-type annotation

We used Seurat (22) to integrate and cluster the collected single cells from COVID19 patients and healthy controls. The gene counts for each cell were normalized by LogNormalize method, which divides gene counts by the total counts for that cell and multiplied by the scale.factor. The normalized gene counts were then natural-log transformed using log1p function. The top 2000 most variable genes were selected using FindVariableFeatures functions for the clustering of single cells. We used dimensions of reduction 30 and resolution 0.3 for the cluster analysis. We used SingleR (23) and human primary cell atlas reference (24) to annotate cell types of single cells. The cell type of the cluster was determined by the dominant cell type in each cluster. The proportion of B cells, T cells, and NK cells in every sample was further calculated to determine the consistency between our data and observed in other published data (25, 26) in the cell type composition difference between severe COVID19 patients and healthy controls.

### Cellular crosstalk analysis

We used iTALK (27) to identify and visualize the possible cellular crosstalk mediated by up-regulated ligand-receptor pairs between each cell type in COVID19 patients. We used the cytokine/chemokine category in the ligand-receptor database for this analysis. We used Wilcoxon rank-sum test to identify the significantly up-regulated genes (adjusted P-value <0.05 & average log fold change >0.1) for every cell type in the severe and mild COVID19 patients, respectively, compared with healthy controls at day 0. We also identified up-regulated genes (adjusted P-value <0.05 & average log fold change >0.1) between day 5 and day 0 in recovered mild COVID19 patients and deceased severe COVID19 patients, respectively. We then matched and paired the up-regulated genes against the ligand-receptor database to construct a putative cell-cell communication network using iTALK. The iTALK defines an interaction score using the log fold change of ligand and receptor to rank these interactions.

### Feature genes and pathways for neutrophils

We collected neutrophil cells from mild and severe COVID19 patients within 72 hours of hospital admission (“T1”), as well as healthy controls to identify significantly up-regulated feature genes (adjusted P-value <0.05 & average log fold change >0.1) by comparing neutrophils from one type (healthy, mild, or severe) to the rest of neutrophils from other types, using cells as biological replicates. The overlap among feature genes identified in healthy, mild, and severe groups was presented in the Venn plot using the Venn package. The top 10 feature genes for each type of neutrophil were presented in a bubble plot using the DotPlot function from the Seurat package. These feature genes were then mapped to human protein-protein interactions (PPIs) downloaded from the BioGRID database (version 4.4.197) using R. The KEGG pathways significantly enriched (adjusted P-value <0.05) in feature genes that connected by PPIs were identified using enrichKEGG function from clusterProfiler package (28). The GSEA analysis was performed using clusterProfiler package (28). The bipartite plot of significant pathways, genes, and PPIs was presented using the Cytoscape tool (29).

### Neutrophil cluster analysis

We integrated neutrophils from mild and severe COVID19 patients, as well as healthy controls to identify neutrophil clusters using resolution 0.5 in Seurat. The significantly up-regulated (adjusted P-value <0.05 & average log fold change >0.1) feature genes for each neutrophil cluster were identified by comparing one cluster to all other clusters. The top 5 feature genes for each cluster were shown in a bubble plot using DotPlot function from the Seurat package. The KEGG and GO pathways significantly enriched (adjusted P-value <0.05) in feature genes were identified for each cluster using clusterProfiler package. The top 3 significant pathways (KEGG) and all significant pathways (GO) were shown in the bubble plot using ggplot2 package. The composition changes of each cluster among healthy control, mild and severe COVID19 were identified using student’s t-test and presented using ggpubr package. We define the severity of COVID19 from 1 to 4, in which 1 means healthy, 2 means mild, 3 means severe but recovered, and 4 means severe patient that deteriorated clinically and later passed away. The association between composition changes of each cluster and severity of COVID19 were identified using spearman’s correlation and the Hmisc package. The significant associations were shown using corrplot package. We further identified differentially expressed genes (DEGs) between severe and mild, mild and healthy respectively, for each cluster that associated with the severity of COVID19. The KEGG pathways significantly enriched (adjusted P-value <0.05) in DEGs were identified using the gseKEGG function from clusterProfiler package. The normalized enrichment score (NES) of significant pathways indicates the activation status of the pathway.

### Statistics

Statistical analysis was performed using R with Student’s t test or analysis of variance (ANOVA). Asterisks on figures indicate statistical significance as follows: *P < 0.05, **P < 0.01, ***P < 0.001, and ****P < 0.0001.

### Study approval and ethics

This study was approved by the Veterans Affairs Ann Arbor Institutional Review Board (IRB) and University of Michigan IRB (IRB-2020-1228 and HUM00181804, respectively). All participants provided written informed consent for sample collection and subsequent analyses. Study procedures adhered to full ethical and safety standards.

## Results

### Neutrophils are major contributors to the inflammatory response relative to other leukocytes during COVID

Activated monocytes and T cells have been portrayed as the primary cellular drivers of inflammation during severe COVID-19. Despite being the predominant leukocyte population in terms of numbers (30), neutrophils have been largely overlooked in human studies due to the inability of these cells to survive long-term storage and cryopreservation. Thus, we performed droplet-based scRNA-seq to examine the transcriptomic profiles of freshly isolated peripheral neutrophils and other leukocytes from hospitalized adult patients with COVID-19 disease and healthy donors (HD) (**Figures S1A,B**). To reduce confounding, we excluded subjects with conditions are known to impact immune responses (Methods). The 11 patients with COVID-19 who met our stringent selection criteria were classified into two groups based upon severity – “mild” (n = 4, hospitalized but needing ≤50% O_2_), or “severe” (n = 7, hospitalized but needing > 50% O_2_ or in the intensive care unit, or ICU). The clinical characteristics of enrolled patients are detailed in **Figure S1B**. Neutrophils and other leukocytes were isolated from peripheral blood samples. Since neutrophils are particularly sensitive to degradation, isolated cells were immediately processed for scRNA-seq experiments (see Methods). Non-neutrophil leukocytes from peripheral blood were included at approximately equal proportions in the scRNA seq analysis to dissect cell-cell interactions.

**Figure 1:**
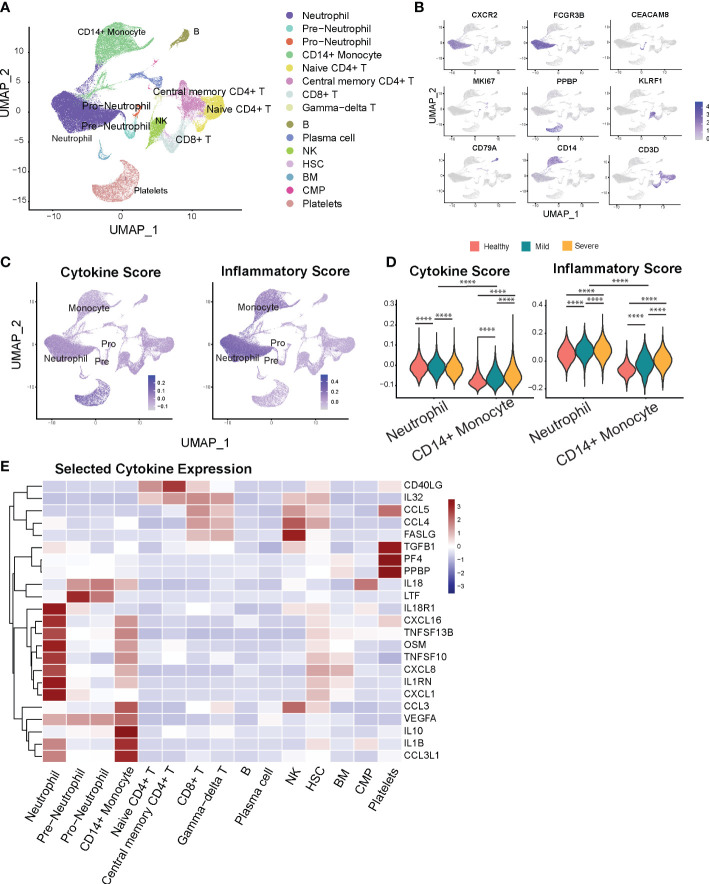
Neutrophils display marked inflammatory signatures relative to other leukocyte populations. **(A)** Cellular populations identified by scRNA seq. The UMAP projection from HD (n = 5), Mild (n = 4), severe (n = 6) samples. **(B)** Canonical cell-defining transcripts were used to label clusters by cell identity as represented in the UMAP plot. Data are colored according to expression levels and the legend is labeled in log scale. **(C)** UMAP plots of cells colored by cytokine score (left) and inflammatory score (right panel). **(D)** Violin plots indicate progression of cytokine (left) and inflammatory scores (right panel) for neutrophils and monocytes with increased severity of infection. **(E)** Heatmap of selected cytokine genes in different subsets of cells. ****p < 0.0001.

A unified single-cell analysis pipeline was employed, including preprocessing involving batch removal and quality control steps (see Methods). A total of 108,597 high-quality cells from all samples proceeded to downstream analysis. Among these cells, 30,429 cells (28%) were from the healthy donor group, 22,188 cells (20%) were from the mild group, and 55,980 cells (52%) were from the severe group. Using Seurat (22) and SingleR (23), we identified 15 major cell types or subtypes according to the expression of canonical gene transcripts in the peripheral blood (**Figures 1A, B**). Among them, 45,463 cells are classified as mature (CXCR2^+^ FCGR3B^+^) or immature (CD24^+^PGLYRP1^+^CEACAM8^+^) neutrophils (**Figures 1A, B**) (7, 31).

Next, we analyzed the transcriptional profiles of the main myeloid (i.e., monocyte and neutrophil) cell populations to determine the differential contributions of each cell type towards the inflammatory landscape during COVID-19. First, to validate the compatibility of our approach with previous studies, we examined transcriptional expression of two monocyte markers most consistently reported to change with COVID-19 severity. Monocytes from severe COVID-19 patients displayed decreased HLA-DRA and increased CD163 expression compared to healthy donors and mild COVID-19 patients (**Figures S2A, B**), consistent with prior reports (10, 15). We next sought to investigate if neutrophils contribute to cytokine storm during COVID-19 disease. To capture a global snapshot of the inflammatory state of different cell populations, we analyzed the cytokine and inflammatory pathways scores in different cell types based on the overall expression of cytokine and inflammatory response genes (**Figures 1C, D**, **Supplementary Table 1**), adapted from (26, 32). Monocytes and megakaryocytes have previously been shown to be major sources of systemic cytokine production based upon this scoring system in COVID-19 patients (26), which we also found in our results. Additionally, we observed that neutrophils have greater potential to contribute to the magnitude of the systemic inflammatory response, indicated by their high cytokine and inflammatory pathway scores (**Figures 1C, D**). In addition, neutrophils outnumber monocytes by 10 to 40-fold in infected subjects (**Figure S2C**). Altogether, the substantially higher numbers and the high inflammatory scores of neutrophils underscore the importance of regulating neutrophil-mediated systemic inflammatory responses in COVID-19.

We then investigated the main inflammatory gene signatures for each leukocyte population and found that neutrophils have a distinct inflammatory cytokine/receptor profile with enrichment of *CXCL1*, *IL1RN*, *CXCL8*, *TNFSF10*, *TNFSF13B*, *CXCL16*, and *IL8R1* (**Figure 1E**). Immature neutrophils express markedly higher levels of lactoferrin (*Ltf*) and *Il18*. Furthermore, we found strong transcriptional upregulation of *S100A9* and *S100A8* alarmins in neutrophils from COVID-19 patients (**Figure S2D**), previously reported to correlate with disease severity (16, 33). This increase persists over time for *S100A8* in the severe compared to the mild COVID-19 group (**Figure S2E**). Together, our data show that relative to other peripheral leukocytes, neutrophils are capable of being a major regulatory cell population that governs the severity and magnitude of the inflammatory response during COVID.

### Identification of dysregulated neutrophil phenotypes in severe COVID-19 patients

**Figure 2:**
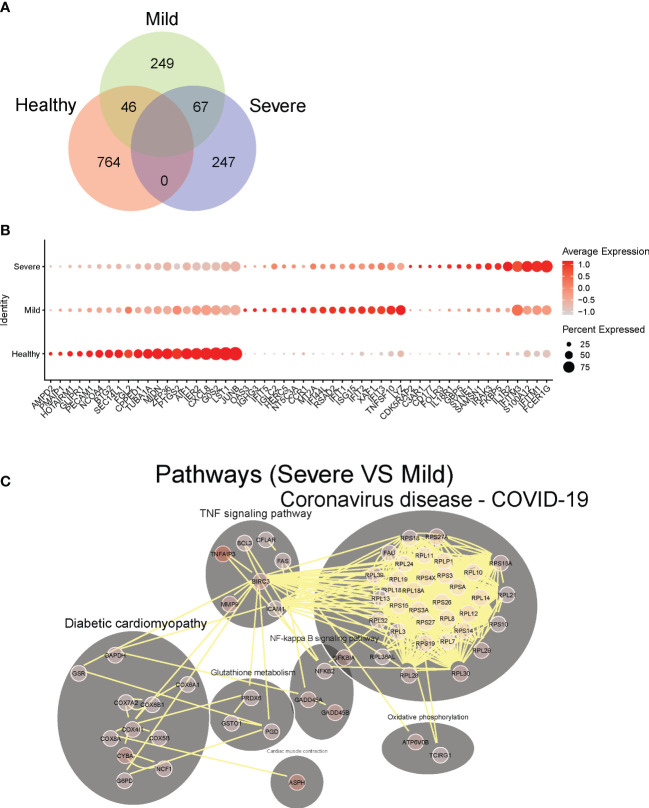
Identification of dysregulated neutrophil phenotypes in severe COVID-19 patients. **(A)** Venn plot of significantly up-regulated genes (adjusted P-value <0.05) in neutrophils from healthy controls, mild and severe COVID19 patients. **(B)** Top 10 differentially expressed upregulated genes in neutrophils from healthy controls, mild and severe COVID19 patients, respectively. **(C)** Predicted cell-cell interaction networks of significantly up-regulated pathways (adjusted P-value <0.05) in neutrophils from severe COVID19 patients compared with that from the mild group.

We next analyzed transcriptional changes within the overall neutrophil population associated with the severity of the disease. Neutrophils from healthy, mild, and severe patient groups show distinct gene expression profiles (**Figure 2A**), reflecting significant transcriptomic changes during disease progression. Neutrophil transcripts which are robustly expressed in the uninfected state, including anti-proliferation and pro-apoptotic genes (*LST1, G0S2, CPPED1, BTG2, PMAIP1*) and anti-inflammatory genes (*AMPD2, SEC14L1*, *ZFP36*), are significantly downregulated in COVID-19 patients (**Figure 2B**). Neutrophils from mild patients have increased expression of genes associated with anti-viral responses, including Interferon stimulated genes (ISGs) and TRAIL (*TNFSF10*) (**Figure 2B**). Remarkably, expression of these genes is attenuated in neutrophils from the severe patients, whose neutrophils displayed increased activation markers including *GBP5* (activator of NLRP3 inflammasome assembly) (34), *FCER1G* (implicated in IL-1b production by neutrophils) (35), and *CD177*, previously associated with COVID-19 severity and death (36), as well as stress-related genes such as *IRAK3, FKBP5, IL1R2* (**Figure 2B**).

To further investigate how neutrophils may functionally differ during infection as compared to healthy controls, we performed pathway analysis of neutrophil transcriptomes. In hospitalized patients with milder COVID-19 disease, we observed broad activation of multiple pathways involved with immune responses to various viral infections, including COVID-19 related pathways (mostly antiviral genes), NOD-like receptor signaling, Toll-like receptor (TLR) signaling, and immune responses to both viral and intracellular pathogens (e.g., influenza A, Salmonella, Epstein-Barr) (**Figure S3**). Thus, upregulated pathways highlight a pronounced diversity of antiviral neutrophil response in hospitalized patients with milder COVID-19. Conversely, in neutrophils from patients with severe disease, we observed significant activation of NF-kB signaling, and TNF signaling pathways, as well as oxidative stress response pathways (e.g., cyclooxygenase genes, glutathione metabolism, and oxidative phosphorylation), compared to those from mild COVID-19 patients (**Figure 2C**). This suggests stress response phenotype in severe patients, rather than a protective-antiviral phenotype seen in the mild disease. Notably, unlike patients with mild disease, severe patients show marked induction of ribosomal genes, suggesting an increase of cellular protein production capacity beyond the observed increase in active gene transcription.

### Neutrophil cell-cell interactions become progressively dysregulated in patients with severe COVID-19

**Figure 3:**
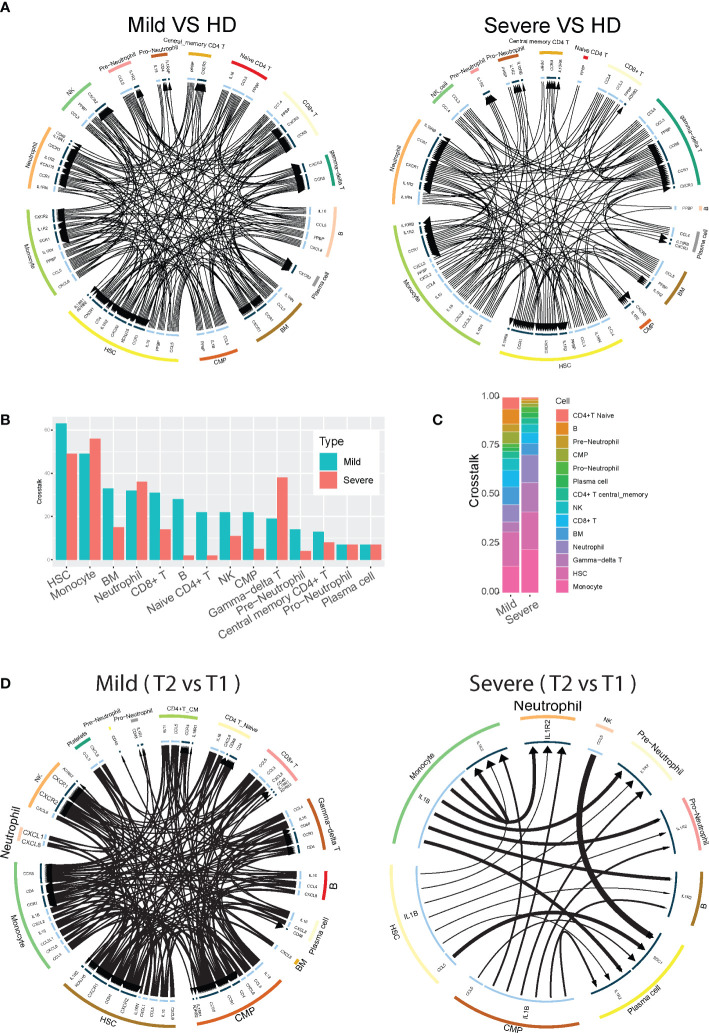
Neutrophil cell-cell interactions become progressively dysregulated in patients with severe COVID-19. **(A)** Circos plot showing the up-regulated cellular crosstalk mediated by significantly (adjusted P-value <0.05) up-regulated ligand-receptor pairs between inflammation-related cell types from mild or severe COVID19 patients compared with that from healthy controls. **(B)** Count of the up-regulated cellular crosstalk for every cell type in mild and severe COVID19 patients, respectively. **(C)** Composition of the up-regulated cellular crosstalk in mild and severe COVID19 patients, respectively. **(D)** Circos plot showing the cellular crosstalk mediated by up-regulated (2nd timepoint versus 1st timepoint, or T2 vs T1) ligand-receptor pairs between inflammation-related cell types from recovered mild (left) and deceased severe (right) COVID19 patients, respectively.

To provide immunologic context for how neutrophils interact with other cell types, we conducted an analysis on the intercellular crosstalk between cytokines and receptors on immune cells. To identify how cytokine-receptor-mediated cell-cell interactions (CCI) evolve across disease severity, we counted the CCIs that represent the active intercellular communication probabilities between up-regulated cytokines and receptors on all cell types in mild versus severe COVID-19 disease. We found that during mild disease, there are overall more active CCIs among all of the different cell populations than that in severe disease (**Figures 3A, B**). Conversely, during severe disease, the number of unique CCIs drop out, resulting in potential degradation of cell-cell cross-regulatory mechanisms. Cell-cell interactions become concentrated and are dominated by interactions between 4 major cell types - neutrophils, monocytes, gamma-delta T cells, and hematopoietic stem cells (HSC), which accounts for more than 60% of the cell-cell interactions in severe disease (**Figure 3C**). As the illness proceeds, we found that in mild patients who recovered from the disease, diverse cell-cell interactions remain preserved at later timepoints, while severe patients who eventually succumb have progressive loss of cell-cell interaction diversity compared to earlier timepoints (**Figure 3D**). These data support a framework where neutrophil cell-cell interactions become progressively dysregulated in patients with severe COVID-19. Additionally, a potential mechanism by which neutrophils contribute to severe inflammation may be reinforcement of activation pathways for specific cell populations such as monocytes and gamma-delta T cells, which have been reported to be robustly pro-inflammatory cell populations during viral infections.

### COVID-19 resulted in alterations of neutrophil subset compositions and their transcription profiles across the levels of the disease severity

**Figure 4:**
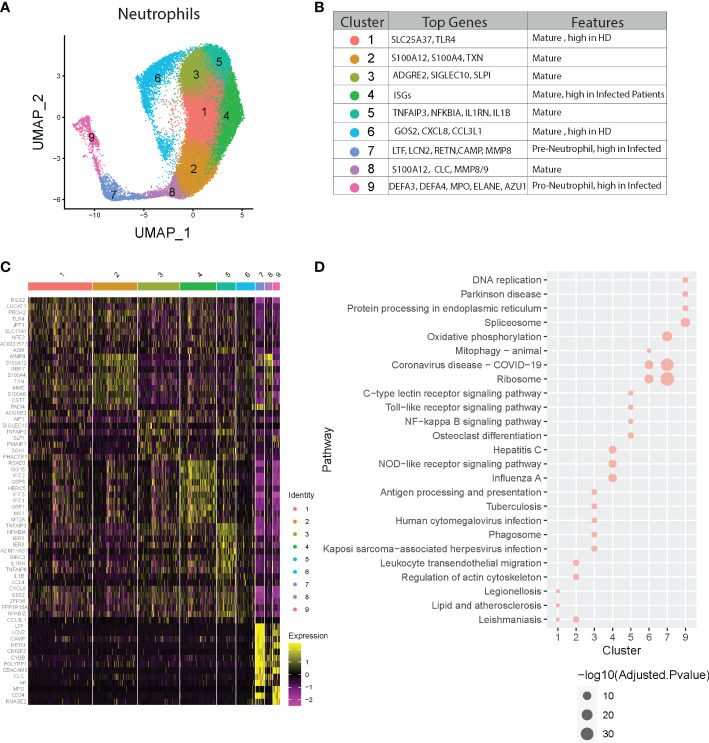
Neutrophil heterogeneity in COVID-19 patients. **(A)** UMAP plot of neutrophil clusters. **(B)** Nomenclature and marker genes for each neutrophil cluster. **(C)** Top5 up-regulated genes in every neutrophil cluster. **(D)** Top3 KEGG pathways significantly enriched in up-regulated genes in every neutrophil cluster.

We next examined whether different phenotypes of neutrophil populations could be identified by scRNA-Seq. We performed cluster analysis of neutrophil scRNA data using the SNN modularity optimization-based clustering algorithm. In total, 9 distinct clusters of neutrophils could be identified based on specific patterns of gene expression. Cluster 9 represents pro-neutrophils (*DEFA3^+^DEFA44^+^MPO^+^ELANE^+^ AZU^+^*; azurophilic granule content genes), cluster 7 represents pre-neutrophils (*LTG^+^LCN2^+^CAMP^+^MMP8* ^+^; specific and gelatinase granule content genes), and the remaining 7 clusters represent mature neutrophils (all *CXCR2* ^+^) (**Figures 4A, B**). The two immature neutrophil clusters (clusters 7 and 9) exhibit robust gene expression of their respective granule content proteins but relative suppression of all of the other genes (**Figure 4C**). Conversely, the mature neutrophil clusters had suppression of granule content genes, but distinct patterns of gene activation that were relatively low in the immature populations (**Figure 4C**). Clusters 2 and 8 displayed upregulation of MMP9, several S100A genes including S100A12, and *MME* (i.e., Neprilysin), which have been implicated in the pathogenesis of COVID-19 (37, 38). Clusters 3, 5, and 6 had high levels of expression of regulatory genes for transcription and apoptosis (**Figure 4C**). Notably, cluster 4 was significantly enriched in interferon (IFN) stimulated genes (ISGs, e.g., *ISG15*, *IFIT* genes, *MX1*, *GBP*, G*BP5*, *HERC5*, and *RSAD2*). Thus, contrary to the assumption that they are a homogenous and transcriptionally quiescent cell population, mature neutrophils display transcriptional diversity with the ability to activate gene expression programs, ranging from immuno-regulatory types to a preferentially antiviral subtype with induction of IFN-stimulated genes.

Subsequent pathway analysis provided insights about possible biological functions of each neutrophil subset (**Figure 4D** and **Figure S4**). Pathway analysis using KEGG and GO of cluster 4 revealed significant activation of viral response pathways as well as NOD-like receptor signaling pathway, supporting its distinct role in anti-viral responses. Other clusters also show specific pathway enrichment; for example, cluster 1 exhibited pathways involved in ferroptosis, cluster 3 and 5 in NF-kappa B signaling, and cluster 7 with activated Ribosome and Coronavirus disease-COVID-19, which is consistent with the concept of “pre-neutrophils” being robustly activated during acute infection with SARS-CoV-2.

**Figure 5:**
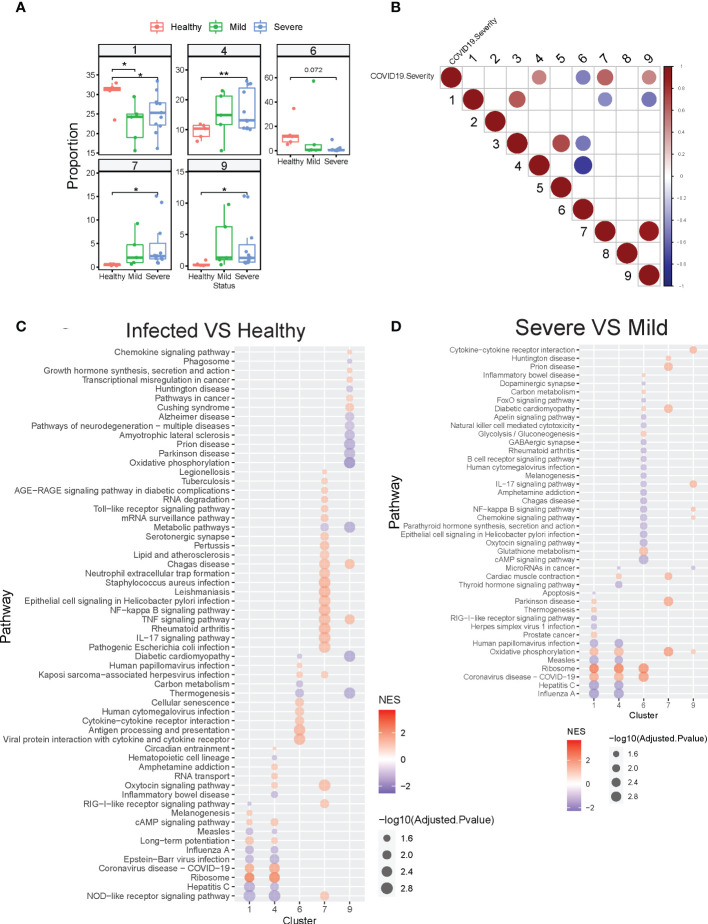
COVID-19 resulted in alterations of neutrophil subset compositions and their transcription profiles. **(A)** Neutrophil clusters that differed in proportions (% of neutrophils) between healthy controls and hospitalized patients with mild or severe COVID19. **(B)** Neutrophil clusters that significantly (adjusted P-value <0.05) associated with the severity of COVID19. Spearman’s’ correlations were used to determine the association between cluster proportion and the severity of COVID19 (healthy 1, mild 2, severe 3, decreased 4). Red: positive correlation; Blue: negative correlation. Depth of color increase with the absolute value of the association. Only significant associations (adjusted P-value <0.05) are shown in this graph. **(C)** GSEA analysis of significantly different KEGG pathway gene sets in selected neutrophil clusters from COVID19 patients compared with that from healthy controls. **(D)** GSEA analysis of significantly different KEGG gene sets in selected neutrophil clusters from severe COVID19 patients compared with that from mild COVID19 patients. *p < 0.05, **p < 0.01. NES, Normalized enrichment score.

Next, we determined whether all of these clusters exist at baseline and whether specific neutrophil subsets were augmented depending on the presence or severity of infection. We found higher proportions of clusters 1 and 6 in healthy compared to infected subjects, while clusters 4, 7, and 9 were increased in COVID-19 patients, especially in the severe group (**Figure 5A**, **Figures S5A, B**). Since clusters 7 and 9 are immature neutrophils, their increase provides evidence of emergency myelopoiesis in severe COVID-19 patients, also supported by previous reports (15, 33). Overall, cluster 4 was significantly associated with disease severity, while cluster 6 was negatively associated with the severity of the disease (**Figure 5B**). Within each cluster, we also observed the neutrophils up-or down-regulating gene groups and pathways based upon disease state (**Figure 5C**, **Figures S6, S7**). For example, compared to healthy controls, cluster 7 neutrophils (immature neutrophils) from infected subjects upregulated genes involved in multiple pathways associated with host defense, including neutrophil extracellular trap formation, cytokine signaling, and NF-kB signaling (**Figure 5C**, **Figure S6**). Cluster 4 neutrophils, which are enriched with anti-viral responses, displayed activation of the ribosome and COVID-19 pathways in patients with mild disease, as compared to healthy controls, with further activation in subjects with severe disease (**Figure 5D**, **Figure S7**). Some pathways involved in anti-viral responses are down-regulated in cluster 4 from severe patients compared with mild patients, consistent with what we discovered in **Figure 2B**. Oxidative phosphorylation pathways were activated in multiple neutrophil clusters in subjects with severe infection compared to those with mild disease (**Figure 5D**). Cluster 6 displayed striking downregulation of multiple pathways during severe disease, including those related to IL-17 signaling, NF-kB, and cAMP signaling (**Figure 5D**). Strikingly, neutrophils displayed progressively decreased activation of hepatitis, influenza, and other viral pathways with increasing COVID-19 disease severity (**Figures 5C, D**). Together, neutrophils heterogeneity and their changes in proportion or transcription are strongly related to the severity of the COVID-19 disease.

## Discussion

Since the start of the SARS-CoV-2 pandemic, a variety of “omic”-based analyses have been utilized to understand the pathogenesis of severe COVID-19 associated infection (7, 31, 39). Notably underrepresented in these studies is a comprehensive analysis of neutrophils, which despite being abundant and widely considered as integral cellular contributors to immune dysregulation, have been largely overlooked for a variety of reasons, including the technical difficulty of isolating neutrophils and preserving them for downstream studies such as single-cell sequencing analyses. Only recently have two groups put forth papers that have explicitly used neutrophil-preserving methods in studying human samples - one using scRNA sequencing-based phenotyping of healthy human subjects, and another examining neutrophil phenotypes from patients with acute respiratory distress syndrome (ARDS) from severe COVID-19 disease or bacterial pneumonia (40, 41). Our work builds upon their findings in that we examined how neutrophil phenotypes differ in relative abundance and pathway activation in patients with mild versus severe COVID-19 disease. We also determined whether all phenotypes exist at baseline, or if new subpopulations of neutrophils emerge during infections, based upon the severity of the infectious insult. Our results suggest that discrete clusters of mature neutrophils exist even under basal uninfected conditions, as reflected by distinct transcriptional profiles and activated pathways. We furthermore observed that the relative proportions of each cluster change during infection and with increasing severity.

Additionally, most scRNA seq studies of patients with COVID have utilized samples collected from subjects that span a large age range (children to elderly) and the full spectrum of co-morbid conditions, which may introduce bias and confounding factors when identifying what mechanisms underlie severe SARS-CoV-2 infections, particularly given the small sample sizes. Our study focused exclusively on human adult patients hospitalized with respiratory manifestations of COVID-19 disease, taking care to exclude subjects with chronic immunosuppression, active malignancy, autoimmune conditions, poorly controlled diabetes, chronic infections, and other advanced co-morbidities that could influence immune responses at baseline. Due to our cohort being mainly Veterans, all of our subjects were males and mostly White. Although the total number of subjects was small, our study was actually one of the largest studies to examine neutrophil responses by scRNA seq. Thus, by controlling for the variability in neutrophil responses that might be introduced by severe chronic comorbidities, sex, age, or race, our results can be considered to reflect the intrinsic heterogeneity of neutrophil responses during health and SARS-CoV-2 infections.

Upon SARS-CoV-2 infection, altered neutrophil abundance and functionality have been linked with the pathophysiology of severe COVID-19 disease (5, 7, 42, 43). We show that neutrophils are a potentially important cellular source of cytokines and can be major contributors to the inflammatory response upon SARS-CoV-2 infection. Importantly, neutrophils display dynamic responses, with evidence of increased oxidative stress and ribosomal pathway activation and suppression of multiple viral pathways (e.g., influenza and measles response pathways) during severe infections. Our results further contribute to our understanding of neutrophil biology, revealing vast heterogeneity and breadth of inflammatory responses in neutrophil subsets in COVID-19 patients (12), in contrast to the prevailing view that neutrophils are a homogeneous antimicrobial cell population.

Our computational clustering revealed extensive heterogeneity in neutrophils with the identification of seven transcriptionally distinctive mature and two immature neutrophil clusters. In particular, the identification of distinct mature neutrophil clusters is an important finding to our understanding of how neutrophils contribute to the pathogenesis of severe infections, as it underscores the importance of recognizing a broader spectrum of neutrophil functional phenotypes. We found those neutrophil subsets display the ability to activate differential gene expression programs, ranging from inhibitory/regulatory subsets to a preferentially antiviral subset with activated IFN-regulated gene expression profile. The proportion of each phenotype correlated with severe disease course. For example, cluster 4 neutrophils showed significantly activated viral response pathways, suggesting a distinct subset of neutrophils in anti-viral responses. However, they also display progressively decreased activation of the viral pathways and increased stress response-related pathways with worsening COVID-19 disease severity. Our findings indicate that neutrophils are capable of mounting effective antiviral defenses but with disease progression, adopt a form of immune dysregulation characterized by excess cellular stress, thereby contributing to the immunopathology of severe SARS-CoV-2 infections.

The progression of ARDS during severe COVID-19 disease, as well as other severe respiratory viral infections, continues despite patients already having cleared the viral infection in the majority of cases, especially in immunocompetent hosts (44). It is during this period when the host is transitioning either to recovery or persistent inflammation that the outcome of infection is determined. The immune mechanisms governing resolution versus persistent inflammation are complex, with evidence of aberrant intercellular regulatory mechanisms that perpetuate inflammation (45). Our work builds upon this concept by examining how systemic neutrophil responses differ in COVID-19 patients, all of whom are sick enough to be hospitalized, but whose respiratory manifestations are milder versus severe. In patients with severe COVID-19, we find evidence of neutrophils no longer acting in concert with other cell types, as reflected by their loss of intensity and diversity of cell-cell interaction with other immune cell populations. To understand how neutrophils might impact systemic inflammatory responses, we found that neutrophils have higher inflammatory scores compared to monocytes, suggesting that they could be a key source of a diverse set of cytokines that are highly elevated in COVID-19 patients with severe disease progression. These findings complement prior reported findings that megakaryocytes and specific monocyte subsets were the primary producers of cytokines (26). Our results indicate that future investigations should identify ways to harness the regulatory capacities of neutrophils in a therapeutic manner, including how to promote antiviral functions early during infection, and perhaps more importantly, how to shift the balance towards a more restorative neutrophil profile as the human host attempts to recover after the infection has been cleared.

Altogether, our report presents details that help us better understand the vast heterogeneity and breadth of inflammatory responses in neutrophil subsets in COVID-19 patients.

## Data availability statement

The datasets presented in this study can be found in online GEO repositories (access number GSE216020).

## Ethics statement

The studies involving human participants were reviewed and approved by University of Michigan IRB. The patients/participants provided their written informed consent to participate in this study.

## Author contributions

The overall project was conceived by MO and JD. JX and BH contributed equally to this work. Order for co-first author was determined by JX’s primary role in manuscript preparation and formatting. Experiments were performed and data were analyzed by JX, BH, LG, and JD. Experimental support and methods: BH, JX, KC, DV, and JD. Substantive writing and revision: JX, BH, LG, MO, and JD. Project supervision: LG, MO, and JD. All authors contributed to the article and approved the submitted version.

## Funding

LG is supported by NIH/NIGMS R01 LM012373, R01 LM012907 awarded by NLM, and R01 HD084633 awarded by NICHD. MO is supported by Veterans Affairs ORD RCS 1IK6 BX003615-01 and Merit BX000656Awards. JD is supported by NIH/NIA R01 AG028082, Veterans Affairs ORD I01BX004565, and I01BX005447.

## Conflict of interest

The authors declare that the research was conducted in the absence of any commercial or financial relationships that could be construed as a potential conflict of interest.

## Publisher’s note

All claims expressed in this article are solely those of the authors and do not necessarily represent those of their affiliated organizations, or those of the publisher, the editors and the reviewers. Any product that may be evaluated in this article, or claim that may be made by its manufacturer, is not guaranteed or endorsed by the publisher.
