# Supplementary material for: Heterogeneity of neutrophils and inflammatory responses in patients with COVID-19 and healthy controls
Source: Front Immunol. 2022 Nov 16;13:970287. doi: 10.3389/fimmu.2022.970287 (PMC9709423; doi:10.3389/fimmu.2022.970287)
Supplement: Supplementary file 1 [file DataSheet_1.pdf]

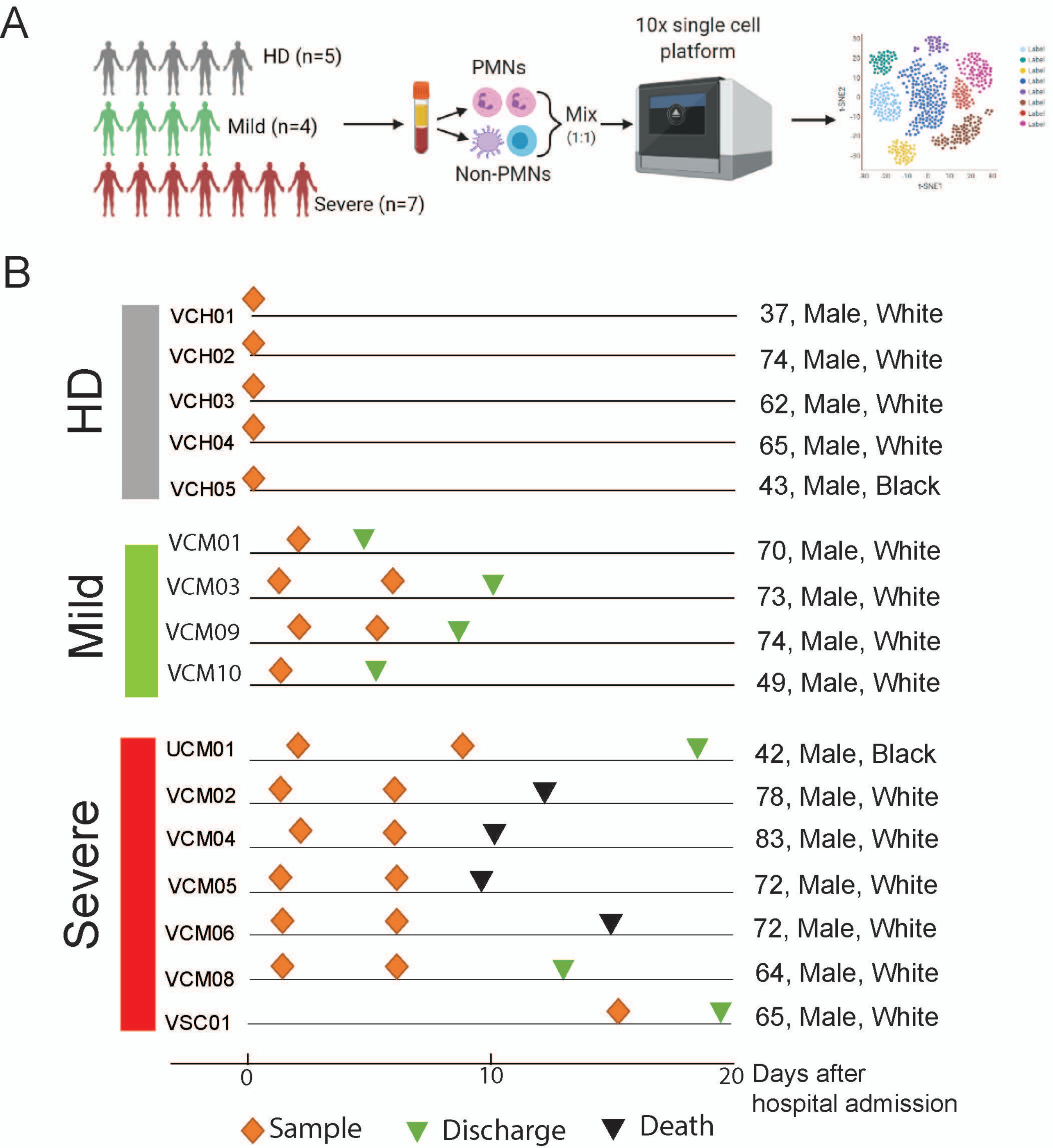

A
HD (n=5)
Mild (n=4)
Severe (n=7)
PMNs
Non-PMNs
Mix
(1:1)
10x single cell
platform
t-SNE1
t-SNE2
B
HD
Mild
Severe
VCH01
VCH02
VCH03
VCH04
VCH05
VCM01
VCM03
VCM09
VCM10
UCM01
VCM02
VCM04
VCM05
VCM06
VCM08
VSC01
37, Male, White
74, Male, White
62, Male, White
65, Male, White
43, Male, Black
70, Male, White
73, Male, White
74, Male, White
49, Male, White
42, Male, Black
78, Male, White
83, Male, White
72, Male, White
72, Male, White
64, Male, White
65, Male, White
0
10
20
Days after
hospital admission
Sample
Discharge
Death

Fig. S1 (A), schematic showing the overall study design. The scRNA-seq was applied to whole blood cells across three conditions and the output data were used for expression analyses. (B), Timeline of the course of disease for 11 patients infected with SARS-CoV-2 enrolled in our study.

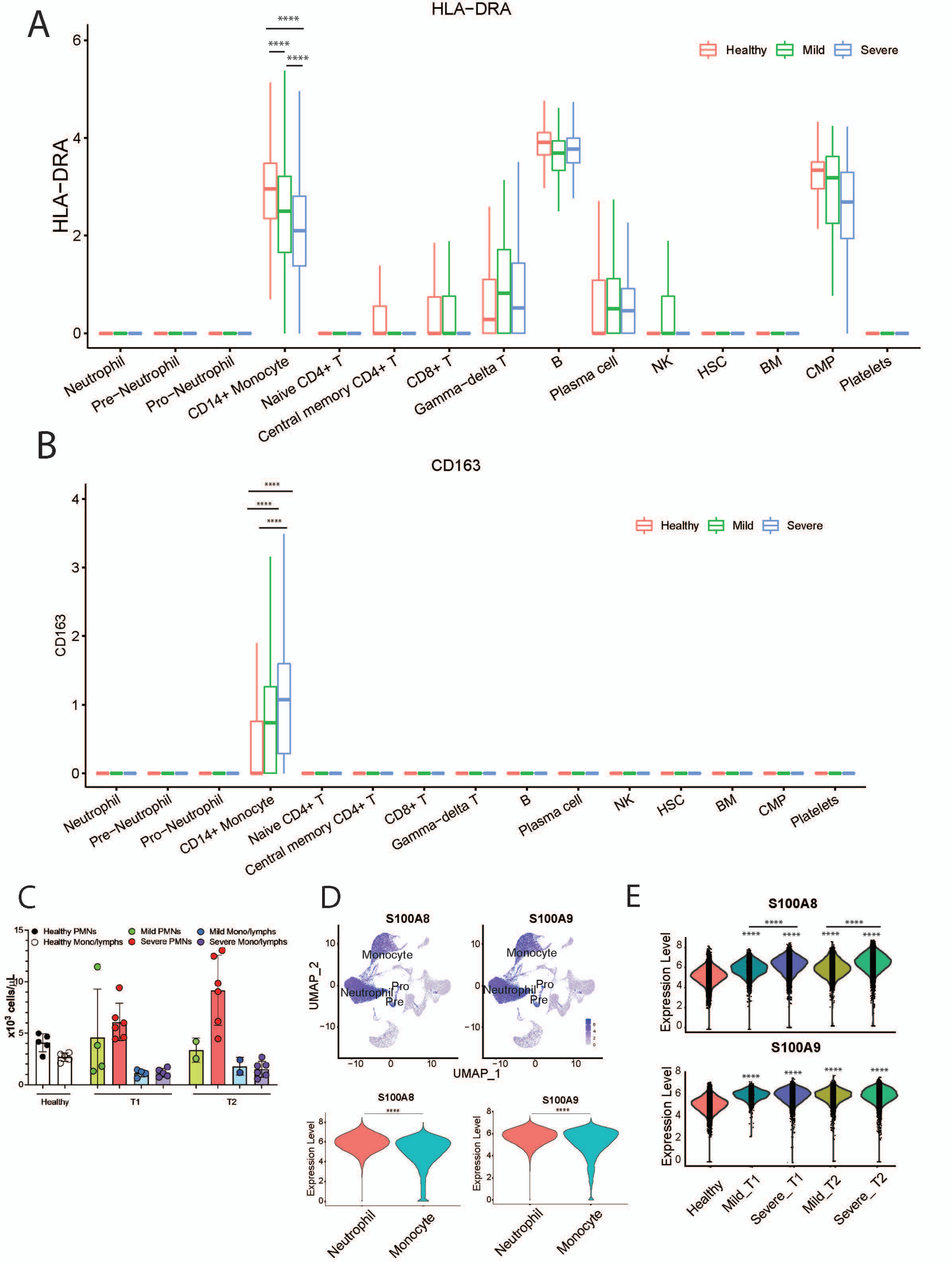
A
HLA-DRA
Healthy
Mild
Severe
****
HLA-DRA
0
2
4
6
Neutrophil
Pre-Neutrophil
Pro-Neutrophil
CD14+ Monocyte
Naive CD4+ T
Central memory CD4+ T
CD8+ T
Gamma-delta T
B
Plasma cell
NK
HSC
BM
CMP
Platelets
B
CD163
CD163
0
1
2
3
4
C
Healthy PMNs
Healthy Mono/lymphs
Mild PMNs
Severe PMNs
Mild Mono/lymphs
Severe Mono/lymphs
x10³ cells/μL
0
5
10
15
Healthy
T1
T2
D
S100A8
S100A9
Monocyte
Neutrophil
Pro
Pre
UMAP_2
UMAP_1
Expression Level
Neutrophil
Monocyte
E
S100A8
S100A9
Expression Level
Healthy
Mild_T1
Severe_T1
Mild_T2
Severe_T2

Fig. S2. (A) Violin plots showing expression of HLA-DRA by different cell clusters. (B) Violin plots showing the expression of CD163 by different cell clusters. (C) Absolute cell number counts in the blood from healthy, mild, and severe patients (D) UMAP plot showing the expression of S100A8 and S100A9 by neutrophils and monocytes. (E) Violin plots showing expression of S100A8 (up) and S100A9 (below) by neutrophils in different groups of patients. Asterisks on figures indicate statistical significance as follows: ****, P < 0.0001.

Fig. S3

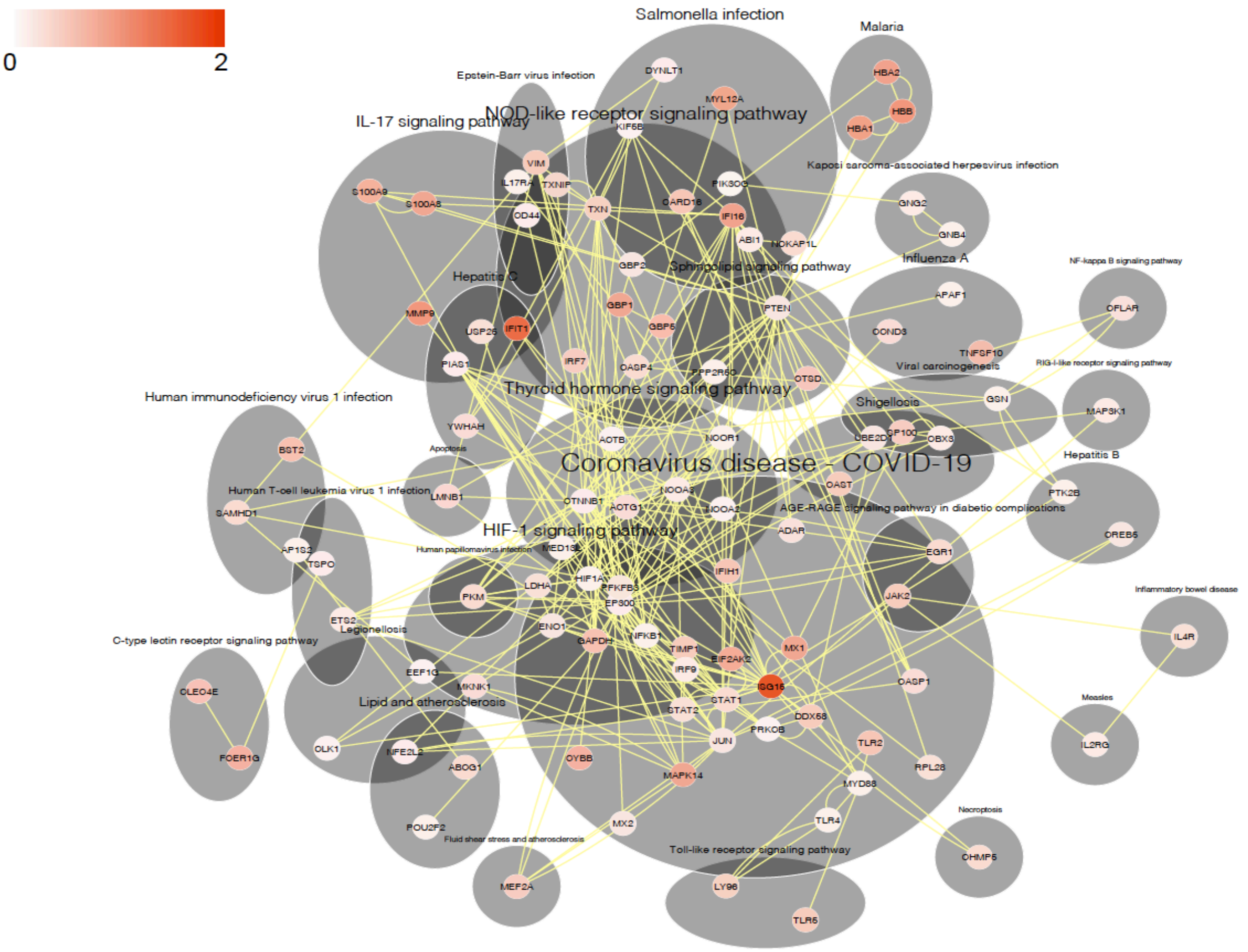

0
2
Salmonella infection
Malaria
Epstein-Barr virus infection
NOD-like receptor signaling pathway
IL-17 signaling pathway
Kaposi sarcoma-associated herpesvirus infection
Influenza A
NF-kappa B signaling pathway
Hepatitis C
Sphingolipid signaling pathway
Viral carcinogenesis
RIG-I-like receptor signaling pathway
Thyroid hormone signaling pathway
Shigellosis
Human immunodeficiency virus 1 infection
Apoptosis
Coronavirus disease - COVID-19
Hepatitis B
Human T-cell leukemia virus 1 infection
AGE-RAGE signaling pathway in diabetic complications
HIF-1 signaling pathway
Human papillomavirus infection
Inflammatory bowel disease
Legionellosis
C-type lectin receptor signaling pathway
Lipid and atherosclerosis
Measles
Necroptosis
Fluid shear stress and atherosclerosis
Toll-like receptor signaling pathway
DYNLT1
MYL12A
HBA2
HBB
HBA1
KIF5B
VIM
IL17RA
TXNIP
CD44
S100A9
S100A8
TXN
PIK3CG
CARD16
IFI16
GNG2
GNB4
ABI1
NCKAP1L
GBP2
APAF1
CFLAR
MMP9
GBP1
PTEN
CCND3
USP25
IFIT1
GBP5
TNFSF10
PIAS1
IRF7
CASP4
PPP2R5C
CTSD
GSN
MAP3K1
YWHAH
ACTB
NCOR1
UBE2D1
SP100
CBX3
BST2
OAS1
PTK2B
LMNB1
CTNNB1
ACTG1
NCOA2
SAMHD1
ADAR
CREB5
AP1S2
TSPO
MED13L
EGR1
HIF1A
LDHA
PKM
PFKFB3
IFIH1
EP300
JAK2
ETS2
ENO1
IL4R
GAPDH
NFKB1
TIMP1
MX1
EIF2AK2
EEF1G
IRF9
ISG15
CLEC4E
MKNK1
OASP1
STAT1
STAT2
DDX58
FCER1G
CLK1
NFE2L2
JUN
PRKCB
TLR2
IL2RG
ABCG1
CYBB
RPL28
MAPK14
MYD88
POU2F2
MX2
TLR4
CHMP5
MEF2A
LY96
TLR5

Fig. S3 Protein-protein interaction networks of significantly up-regulated pathways (adjusted P-value <0.05) in neutrophils from mild COVID19 patients compared with that from healthy controls. Color bar indicates fold change of genes between groups.

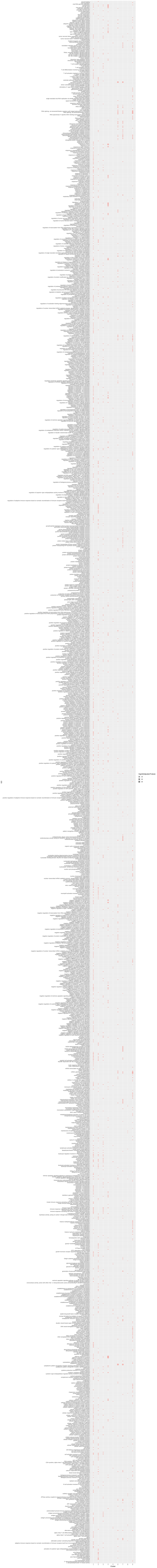

−log10(Adjusted.Pvalue)
10
20
30

Fig. S4 GO pathways significantly enriched in up-regulated genes in every neutrophil cluster.

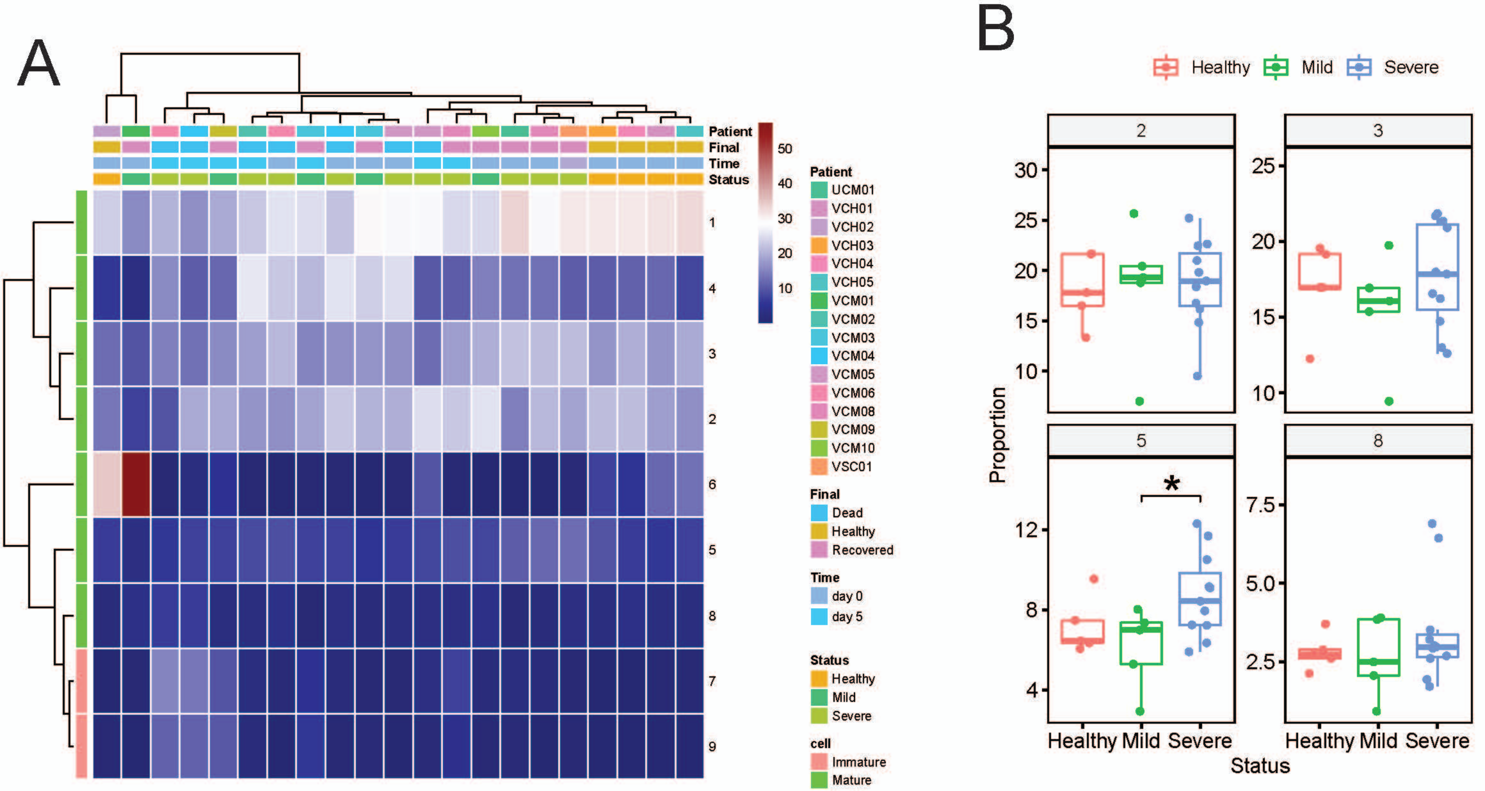
A
B
Patient
Final
Time
Status
1
4
3
2
6
5
8
7
9
50
40
30
20
10
Patient
UCM01
VCH01
VCH02
VCH03
VCH04
VCH05
VCM01
VCM02
VCM03
VCM04
VCM05
VCM06
VCM08
VCM09
VCM10
VSC01
Final
Dead
Healthy
Recovered
Time
day 0
day 5
Status
Healthy
Mild
Severe
cell
Immature
Mature
Healthy
Mild
Severe
2
3
5
8
*
Proportion
Status

Fig. S5 (A) Heatmap of neutrophil cluster size in every sample. (B) Proportional change of neutrophil clusters during COVID19 progression. * $p < 0.05$

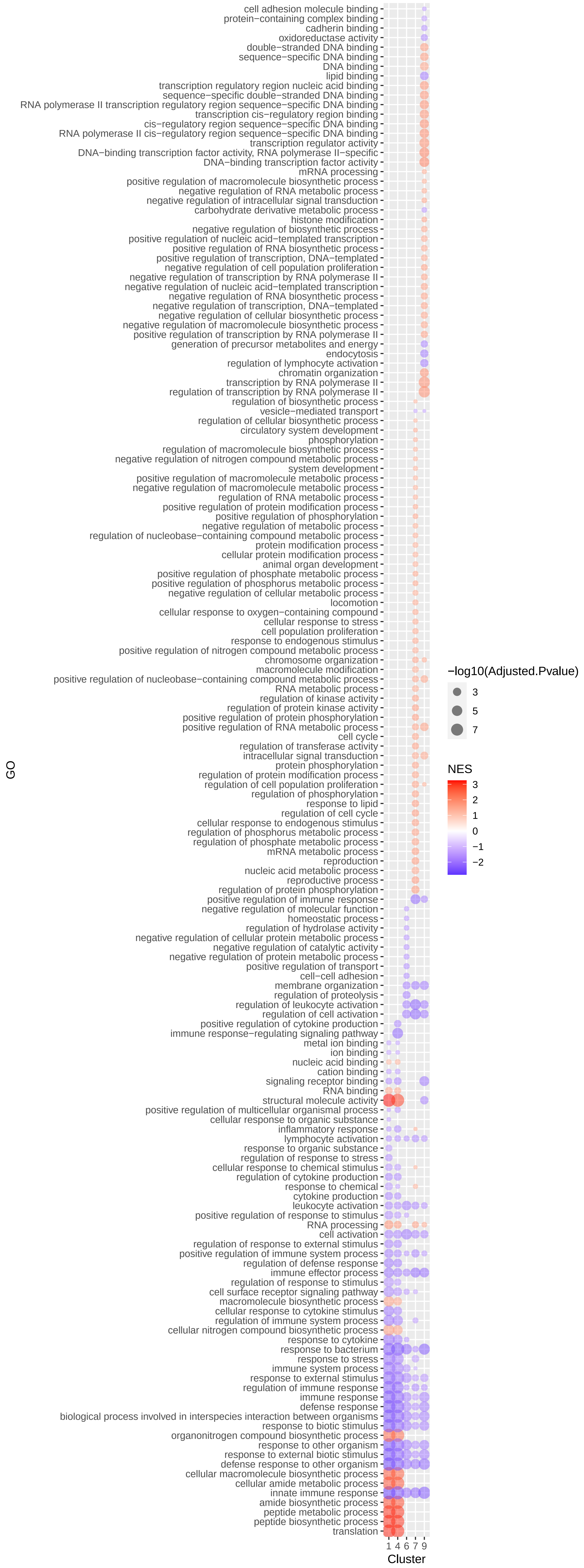
cell adhesion molecule binding
protein−containing complex binding
cadherin binding
oxidoreductase activity
double−stranded DNA binding
sequence−specific DNA binding
DNA binding
lipid binding
transcription regulatory region nucleic acid binding
sequence−specific double−stranded DNA binding
RNA polymerase II transcription regulatory region sequence−specific DNA binding
transcription cis−regulatory region binding
cis−regulatory region sequence−specific DNA binding
RNA polymerase II cis−regulatory region sequence−specific DNA binding
transcription regulator activity
DNA−binding transcription factor activity, RNA polymerase II−specific
DNA−binding transcription factor activity
mRNA processing
positive regulation of macromolecule biosynthetic process
negative regulation of RNA metabolic process
negative regulation of intracellular signal transduction
carbohydrate derivative metabolic process
histone modification
negative regulation of biosynthetic process
positive regulation of nucleic acid−templated transcription
positive regulation of RNA biosynthetic process
positive regulation of transcription, DNA−templated
negative regulation of cell population proliferation
negative regulation of transcription by RNA polymerase II
negative regulation of nucleic acid−templated transcription
negative regulation of RNA biosynthetic process
negative regulation of transcription, DNA−templated
negative regulation of cellular biosynthetic process
negative regulation of macromolecule biosynthetic process
positive regulation of transcription by RNA polymerase II
generation of precursor metabolites and energy
endocytosis
regulation of lymphocyte activation
chromatin organization
transcription by RNA polymerase II
regulation of transcription by RNA polymerase II
regulation of biosynthetic process
vesicle−mediated transport
regulation of cellular biosynthetic process
circulatory system development
phosphorylation
regulation of macromolecule biosynthetic process
negative regulation of nitrogen compound metabolic process
system development
positive regulation of macromolecule metabolic process
negative regulation of macromolecule metabolic process
regulation of RNA metabolic process
positive regulation of protein modification process
positive regulation of phosphorylation
negative regulation of metabolic process
regulation of nucleobase−containing compound metabolic process
protein modification process
cellular protein modification process
animal organ development
positive regulation of phosphate metabolic process
positive regulation of phosphorus metabolic process
negative regulation of cellular metabolic process
locomotion
cellular response to oxygen−containing compound
cellular response to stress
cell population proliferation
response to endogenous stimulus
positive regulation of nitrogen compound metabolic process
chromosome organization
macromolecule modification
positive regulation of nucleobase−containing compound metabolic process
RNA metabolic process
regulation of kinase activity
regulation of protein kinase activity
positive regulation of protein phosphorylation
positive regulation of RNA metabolic process
cell cycle
regulation of transferase activity
intracellular signal transduction
protein phosphorylation
regulation of protein modification process
regulation of cell population proliferation
regulation of phosphorylation
response to lipid
regulation of cell cycle
cellular response to endogenous stimulus
regulation of phosphorus metabolic process
regulation of phosphate metabolic process
mRNA metabolic process
reproduction
nucleic acid metabolic process
reproductive process
regulation of protein phosphorylation
positive regulation of immune response
negative regulation of molecular function
homeostatic process
regulation of hydrolase activity
negative regulation of cellular protein metabolic process
negative regulation of catalytic activity
negative regulation of protein metabolic process
positive regulation of transport
cell−cell adhesion
membrane organization
regulation of proteolysis
regulation of leukocyte activation
regulation of cell activation
positive regulation of cytokine production
immune response−regulating signaling pathway
metal ion binding
ion binding
nucleic acid binding
cation binding
signaling receptor binding
RNA binding
structural molecule activity
positive regulation of multicellular organismal process
cellular response to organic substance
inflammatory response
lymphocyte activation
response to organic substance
regulation of response to stress
cellular response to chemical stimulus
regulation of cytokine production
response to chemical
cytokine production
leukocyte activation
positive regulation of response to stimulus
RNA processing
cell activation
regulation of response to external stimulus
positive regulation of immune system process
regulation of defense response
immune effector process
regulation of response to stimulus
cell surface receptor signaling pathway
macromolecule biosynthetic process
cellular response to cytokine stimulus
regulation of immune system process
cellular nitrogen compound biosynthetic process
response to cytokine
response to bacterium
response to stress
immune system process
response to external stimulus
regulation of immune response
immune response
defense response
biological process involved in interspecies interaction between organisms
response to biotic stimulus
organonitrogen compound biosynthetic process
response to other organism
response to external biotic stimulus
defense response to other organism
cellular macromolecule biosynthetic process
cellular amide metabolic process
innate immune response
amide biosynthetic process
peptide metabolic process
peptide biosynthetic process
translation
GO
1 4 6 7 9
Cluster
−log10(Adjusted.Pvalue)
3
5
7
NES
3
2
1
0
−1
−2

Fig. S6 GSEA analysis of significantly different GO pathway gene sets in selected neutrophil clusters from COVID19 patients compared with that from healthy controls. NES, normalized enrichment score.

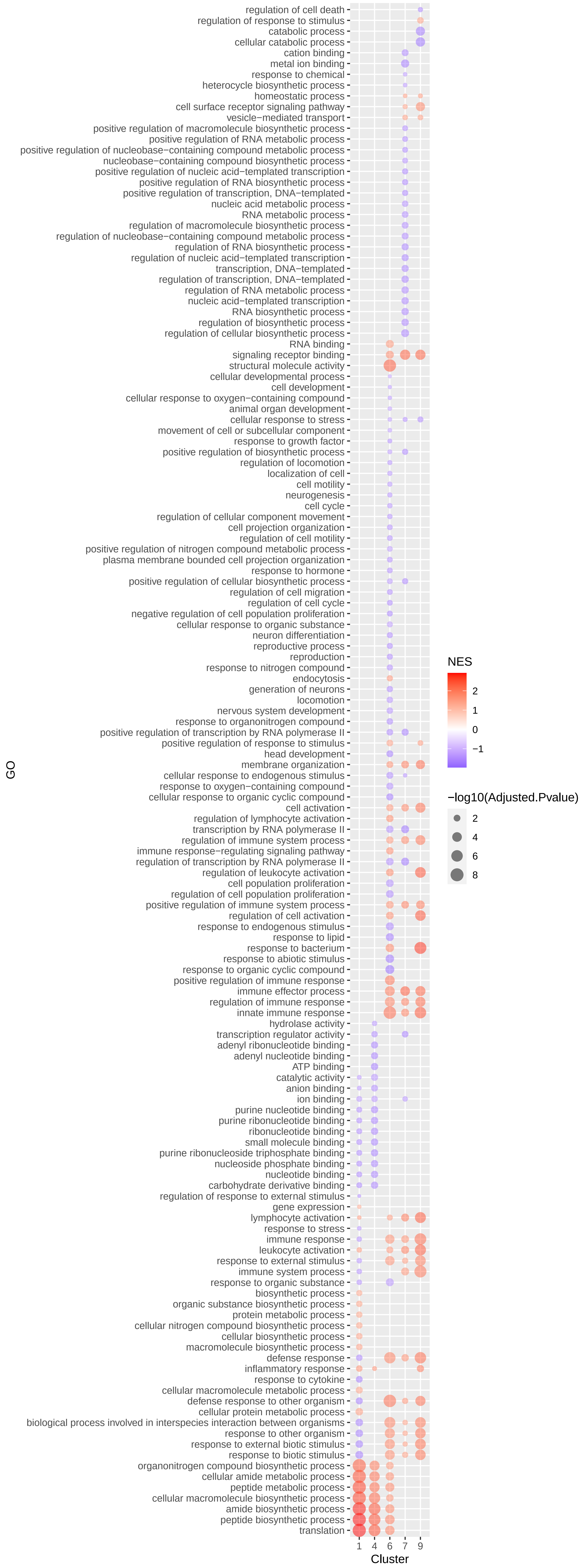

regulation of cell death
regulation of response to stimulus
catabolic process
cellular catabolic process
cation binding
metal ion binding
response to chemical
heterocycle biosynthetic process
homeostatic process
cell surface receptor signaling pathway
vesicle−mediated transport
positive regulation of macromolecule biosynthetic process
positive regulation of RNA metabolic process
positive regulation of nucleobase−containing compound metabolic process
nucleobase−containing compound biosynthetic process
positive regulation of nucleic acid−templated transcription
positive regulation of RNA biosynthetic process
positive regulation of transcription, DNA−templated
nucleic acid metabolic process
RNA metabolic process
regulation of macromolecule biosynthetic process
regulation of nucleobase−containing compound metabolic process
regulation of RNA biosynthetic process
regulation of nucleic acid−templated transcription
transcription, DNA−templated
regulation of transcription, DNA−templated
regulation of RNA metabolic process
nucleic acid−templated transcription
RNA biosynthetic process
regulation of biosynthetic process
regulation of cellular biosynthetic process
RNA binding
signaling receptor binding
structural molecule activity
cellular developmental process
cell development
cellular response to oxygen−containing compound
animal organ development
cellular response to stress
movement of cell or subcellular component
response to growth factor
positive regulation of biosynthetic process
regulation of locomotion
localization of cell
cell motility
neurogenesis
cell cycle
regulation of cellular component movement
cell projection organization
regulation of cell motility
positive regulation of nitrogen compound metabolic process
plasma membrane bounded cell projection organization
response to hormone
positive regulation of cellular biosynthetic process
regulation of cell migration
regulation of cell cycle
negative regulation of cell population proliferation
cellular response to organic substance
neuron differentiation
reproductive process
reproduction
response to nitrogen compound
endocytosis
generation of neurons
locomotion
nervous system development
response to organonitrogen compound
positive regulation of transcription by RNA polymerase II
positive regulation of response to stimulus
head development
membrane organization
cellular response to endogenous stimulus
response to oxygen−containing compound
cellular response to organic cyclic compound
cell activation
regulation of lymphocyte activation
transcription by RNA polymerase II
regulation of immune system process
immune response−regulating signaling pathway
regulation of transcription by RNA polymerase II
regulation of leukocyte activation
cell population proliferation
regulation of cell population proliferation
positive regulation of immune system process
regulation of cell activation
response to endogenous stimulus
response to lipid
response to bacterium
response to abiotic stimulus
response to organic cyclic compound
positive regulation of immune response
immune effector process
regulation of immune response
innate immune response
hydrolase activity
transcription regulator activity
adenyl ribonucleotide binding
adenyl nucleotide binding
ATP binding
catalytic activity
anion binding
ion binding
purine nucleotide binding
purine ribonucleotide binding
ribonucleotide binding
small molecule binding
purine ribonucleoside triphosphate binding
nucleoside phosphate binding
nucleotide binding
carbohydrate derivative binding
regulation of response to external stimulus
gene expression
lymphocyte activation
response to stress
immune response
leukocyte activation
response to external stimulus
immune system process
response to organic substance
biosynthetic process
organic substance biosynthetic process
protein metabolic process
cellular nitrogen compound biosynthetic process
cellular biosynthetic process
macromolecule biosynthetic process
defense response
inflammatory response
response to cytokine
cellular macromolecule metabolic process
defense response to other organism
cellular protein metabolic process
biological process involved in interspecies interaction between organisms
response to other organism
response to external biotic stimulus
response to biotic stimulus
organonitrogen compound biosynthetic process
cellular amide metabolic process
peptide metabolic process
cellular macromolecule biosynthetic process
amide biosynthetic process
peptide biosynthetic process
translation
GO
1
4
6
7
9
Cluster
NES
2
1
0
−1
−log10(Adjusted.Pvalue)
2
4
6
8

Fig. S7 GSEA analysis of significantly different GO gene sets in selected neutrophil clusters from severe COVID19 patients compared with that from mild COVID19 patients.
